# Draft Genome Sequence of *Pseudoalteromonas* sp. Strain PAB 2.2 Isolated from Abrolhos Bank (Brazil)

**DOI:** 10.1128/genomeA.00016-17

**Published:** 2017-03-09

**Authors:** Bruno S. O. Silva, Maria S. Nobrega, Luciana Leomil, Diogo A. Tschoeke, Gizele D. Garcia, Graciela Dias, Cristiane C. Thompson, Fabiano L. Thompson

**Affiliations:** a Instituto de Biologia, Universidade Federal do Rio de Janeiro, Rio de Janeiro, Brazil; b Núcleo em Ecologia e Desenvolvimento Sócio-Ambiental de Macaé (NUPEM), Universidade Federal do Rio de Janeiro, Macaé, Rio de Janeiro, Brazil; c Instituto de Microbiologia, Universidade Federal do Rio de Janeiro, Macaé, Rio de Janeiro, Brazil; d SAGE, COPPE, Federal University of Rio de Janeiro, Rio de Janeiro, Brazil

## Abstract

We present here the draft genome sequence of *Pseudoalteromonas* sp. strain PAB 2.2, isolated from water of Parcel de Abrolhos coral reef (17°57′32.7″; 38°30′20.3″), on Abrolhos Bank, at a depth of 12 m. The assembly consists of 4,434,635 bp and contains 40 contigs, with a G+C content of 41.60%.

## GENOME ANNOUNCEMENT

The genus *Pseudoalteromonas* was described in 1995, when it was separated from the genus *Alteromonas* (1) and includes Gram-negative, gammaproteobacteria, heterotrophic and aerobic with polar flagellum bacteria. *Pseudoalteromonas* spp. are involved in the production of antimicrobial metabolites that protect coral holobionts against pathogens (2) and show the ability to degrade many components of petroleum (3). *Pseudoalteromonas* spp. can be found in a variety of habitats, including deep and surface waters (4, 5), polar waters (6), and sediments (7). These distinct lifestyles are based on different sets of genes, such as those for lateral flagellum expression in sediment strains and those for reactive oxygen production in polar strains (6, 7). These ubiquitous characteristics point to expressive and diverse adaptive strategies for *Pseudoalteromonas* spp., leading to important research topics. Here, we present the genome sequence of *Pseudoalteromonas* sp. strain PAB2.2, which was isolated from the waters of Parcel de Abrolhos coral reef, in Abrolhos Bank, at a depth of 12 m.

The DNA was extracted using an adaptation of Pitcher's protocol (8). The genomic DNA was sequenced using a Nextera XT DNA sample preparation kit (Illumina, San Diego, CA, USA). The size distribution of the libraries was evaluated using a 2100 Bioanalyzer and a High-Sensitivity DNA kit (Agilent, Santa Clara, CA, USA). A 7500 Real Time PCR machine (Applied Biosystems, Foster City, CA, USA) and a KAPA Library Quantiﬁcation kit (Kapa Biosystems, Wilmington, MA, USA) were used for the quantification of the libraries. Paired-end sequencing (2 × 300 bp) was performed on a MiSeq platform (Illumina, Rio de Janeiro, Brazil). The sequences obtained were preprocessed using PRINSEQ software to remove reads smaller than 35 bp and sequences with a Phred score lower than 30 (9). Sequence reads were assembled using A5-Miseq software (10) with default parameters. A second assembly using CAP3 software (11), based on the contigs obtained, was completed to improve the assembly, as done previously (12). The gene prediction and functional annotation were performed using the RAST server (13).

The sequencing generated 40 contigs that concatenate in 4,434,635 bp with a G+C content of 41.60%. RAST predicted 2,926 open reading frames, 3,926 coding sequences, and 115 RNA sequences (100 tRNAs and 15 rRNAs). Analyzing the genes predicted by RAST, it was possible to observe 95 genes involved in resistance to antibiotics and toxic compounds, such as multidrug resistance efflux pumps and copper, zinc, and arsenic resistance; 11 genes for the metabolism of aromatic compounds, especially benzoate degradation; and 126 genes for motility and chemotaxis, including operons *fli*, *flg*, and *flh* and genes for flagellar motor rotation proteins MotA, MotX, MotB, and MotY. The absence of genes encoding for lateral flagellum indicates that even though this strain presents a great number of genes related to motility, it is incapable of surviving on sediments.

### Accession number(s).

This whole-genome shotgun project has been deposited in GenBank under the accession number LYPI00000000. The version described in this paper is the first version.
